# Mental health of displaced and returnee populations: Insight from the Sri Lankan post-conflict experience

**DOI:** 10.1186/s13031-015-0049-2

**Published:** 2015-08-06

**Authors:** Chesmal Siriwardhana

**Affiliations:** Faculty of Medical Science, Anglia Ruskin University, Chelmsford, CM1 1SQ UK; Institute of Psychiatry, King’s College London, London, UK; Institute for Research & Development, Sri Jayawardenepura Kotte, Sri Lanka

## Abstract

The month of May 2015 marked the sixth year since the end of conflict in Sri Lanka. The cause of death, destruction and displacement, three decades of conflict has had a major impact on health, especially on mental health of those affected by forced displacement. Post-conflict regions of Sri Lanka has seen improvements in many areas, including resettlement of displaced populations and rebuilding of health-related infrastructure. However, substantial gaps exist around the management of health needs among returnee populations, especially in the area of psychosocial health. Long-term mental health and resilience trajectories of those affected by prolonged displacement and experiencing return migration during post-conflict periods remain important, yet critically understudied areas.

The number of internally displaced persons (IDPs) is growing exponentially in many global regions. Conflicts in the Middle East, Africa and Eastern Europe have contributed to population groups being displaced at alarming rates, making humanitarian support provision extremely difficult. The Internal Displacement Monitoring Centre (IDMC), in its Global Overview released in May 2015, reported the highest ever number of IDPs on their record for the year 2014, with a staggering 38 million IDPs globally due to conflict [1]. However, in somewhat lesser numbers, IDPs are also engaged in a return migration process in several post-conflict regions in Africa and Asia. Internal displacement has been strongly linked to poor mental health among IDPs, especially in low and middle income countries [2]. However, studies on mental health of returnee IDPs are limited.

In May 2009, six years ago, Sri Lanka's brutal three decade civil conflict came to an end. During its course, this conflict based on ethnic lines had caused the death of over 100,000 people and displaced many hundreds of thousands, mainly in the Northern and Eastern provinces [3, 4]. It had taken an immeasurable toll on Sri Lanka's economy, society, culture, and crucially, the health of its people. IDPs, some of them forcibly displaced over two decades ago, have borne the brunt of conflict-related physical and psychosocial ill health. Although comprehensive data on physical/mental health burden from former conflict regions are unavailable, some recent studies in particular have shed light on mental health issues among IDPs, especially on those who are returning to areas of origin after conflict [5–7].

Findings from a recent study with IDPs and returning IDPs in Sri Lanka and the value of such studies in improving the understanding about the needs of IDPs are discussed here. ‘COmmon Mental Disorders and Resilience Among Internally Displaced and Return Migrants in Sri Lanka (COMRAID/COMRAID-R)’ was a two-phase study conducted in 2011 and 2012 is the first comprehensive investigation on prolonged forced displacement, return migration and associated mental health impact in Sri Lanka [5, 6]. Key objectives of the study were to describe the prevalence of common mental disorders (CMD; depression, anxiety, PTSD), investigate associations between CMD-socio-economic factors and to examine individual resilience, social support and social networks among a group of IDPs displaced from northern Sri Lanka in 1990, who, after a period of prolonged displacement, had started the return migration process following conflict cessation in 2009.

In addition to CMD prevalence and socio-demographic associations studied at two separate time points, the study also looked into dynamics of resilience and associations with mental disorder outcomes [7]. CMD prevalence measured at baseline in 2011 (18.8 %) was found to be higher than the national average (11.7 %) [5]. Follow-up was conducted in 2012, one year after the baseline phase, and three years after the end of conflict. Methodology/measurements were similar to baseline, and CMD prevalence showed more than a twofold reduction (from 18.8 to 8.6 %) [5]. This reduction could not be explained by any methodological factors and points more towards a general sense of hope and security among the IDP community after the end of conflict. Resilience levels were higher at the follow-up stage and associations were observed with factors such as food insecurity and social isolation/support [7].

Six years have elapsed since the end of conflict in Sri Lanka. There are varying estimates on remaining IDP numbers (IDMC figures around 90000, official government figures at 24000) [1, 8]. The Northern Muslims who had been displaced by conflict in 1990 and spent over 20 years in displacement are no longer considered as IDPs as full return has been supported, promoted and achieved according to official government policy. Other IDPs, mainly Tamil populations displaced during the last stages of conflict have also returned from temporary camps. Although the Sri Lankan IDP situation has improved and significant advances can be noted around legislation and policy overseeing return migration, rehabilitation and rebuilding in former conflict areas, key gaps exist in IDP management approaches, frameworks and policy platforms. Regrettably, a sizeable decline of funding for development, health, psychosocial support and capacity building programmes carried out by international agencies has been observed.

Findings from COMRAID/COMRAID-R are important in the context of post-conflict management of IDPs and returning migrants, and has already contributed to developing interventions on integrating mental health into primary care in post-conflict regions of Sri Lanka. Longitudinal dynamics of resilience and associations with mental disorders have not received sufficient attention and COMRAID/COMRAID-R studies are some of the global firsts to explore these areas, relevant in developing psychosocial interventions for returning forced migrant populations.

The COMRAID-R follow-up phase was a unique opportunity to explore mental health of returning IDPs after prolonged displacement, an understudied area in global literature [9]. Studying populations in prolonged displacement or returnees (especially mental health research) presents distinctive conceptual, methodological and logistical challenges. These challenges may be linked to geopolitical situations beyond the control of researchers, methodological issues around complex causalities of displacement/return and difficulties in conceptualising displacement/return in varied regional, cultural or conflict-specific contexts, logistical challenges of following/finding returning IDPs over time in home areas, and may possibly explain the lack of research in these domains [2, 9–11]. The Sri Lankan COMRAID/COMRAID-R studies has faced and overcome a number of critical challenges stemming from rapidly changing ground realities in post-conflict regions of Sri Lanka, complex population structure and sampling, issues around participant follow-up, ethical issues, cultural issues, complexity of the conflict and post-conflict political landscape [5–7, 12]. COMRAID/COMRAID-R studies therefore provide valuable insight on overcoming research challenges in resource-poor settings.

The return migration process requires a better understanding, especially on factors that may impact on the mental health of returnees. Post-conflict populations in Sri Lanka and elsewhere would benefit from studies with a longitudinal focus that enables the understanding of mental health trajectories after the return process. Studies are required to look at specific sub groups within IDP communities, such as women, children and especially those of old age, to understand the impact of prolonged displacement and return migration on mental health in vulnerable populations. Current global studies on returning IDPs are mostly development-focused and lack wider health orientation, and mental health studies are scarce. Health and wellbeing issues are as important as the development-related issues of food, safety, land, infrastructure and sustainable living for returning IDPs in post-conflict areas. Association of factors such as female gender, unemployment and food insecurity with mental health, as identified in COMRAID/COMRAID-R, is also likely to be strongly linked to development.

Sri Lanka's public health system, long hailed as a success story, has coped relatively well with myriad health issues stemming from 30 years of conflict [4]. Although health service provision in former conflict areas have vastly improved, there are still significant gaps in mental health care. While Sri Lanka recognizes the need to provide adequate health care for IDPs, the 'right to health' may sometimes be overlooked as precedence is given to developmental goals. However, all actors involved in the healthcare provision and management of IDP in Sri Lanka including the government, health sector, international and local humanitarian agencies need to realize the potential longitudinal effects of displacement and the possible negative impact on mental health.

Addressing mental health needs of conflict-affected populations requires a combined effort from humanitarian agencies, governments, clinicians, public health professionals and researchers, and needs to be firmly based on a broad public health approach, rather than a traditional psychiatric care approach. Progressive research that looks beyond the 'trauma model' and at broader mental health consequences is needed in order to develop effective and viable interventions for these populations. Despite circumstantial differences with global IDP populations, the Sri Lankan findings will be useful in providing mental health care to IDPs in other post-conflict countries, as well as for those displaced by current conflicts.
